# Expression of matrix metalloproteinase 2 and 9 in breast cancer and breast fibroadenoma: a randomized, double-blind study

**DOI:** 10.18632/oncotarget.27347

**Published:** 2019-12-03

**Authors:** Luana Mota Martins, Carla Solange de Melo Escorcio Dourado, Larysse Maira Campos-Verdes, Fabiane Araújo Sampaio, Camila Maria Simplício Revoredo, Danylo Rafhael Costa-Silva, Maria da Conceição Barros-Oliveira, Elmo de Jesus Nery Junior, Lucia Maria do Rego-Medeiros, Luiz Henrique Gebrim, Francisco Adelton Alves-Ribeiro, Gilmara Péres Rodrigues, Diego Cipriano Chagas, Dilina do Nascimento Marreiro, Benedito Borges da Silva

**Affiliations:** ^1^Postgraduate Program, Northeast Biotechnology Network (RENORBIO), Biotechnology in Health, Federal University of Piaui, Teresina, Piaui 64000-020, Brazil; ^2^Facid / Wyden Differential Integral Medicine Faculty, Department of Mastology, Teresina, Piaui 64052-810, Brazill

**Keywords:** breast neoplasms, fibroadenoma, matrix metalloproteinase-2, matrix metalloproteinase-9, immunohistochemistry

## Abstract

**Background:**

Matrix metalloproteinases (MMPs) 2 and 9 may play an important role in cell proliferation and dissemination of cancer. However, few studies have compared the expression of these proteins between breast cancer and fibroadenoma.

**Material and methods:**

A randomized, double-blind study was carried out in 66 premenopausal women, aged 20-49 years, who had been diagnosed with fibroadenoma or breast cancer. The patients were divided into two groups: Group A, control (fibroadenoma, n=36) and Group B, study (cancer, n=30). Immunohistochemical analysis was performed using tissue samples of fibroadenoma and breast cancer to assess MMP-2 and MMP-9 antigen expression. Cells were considered positive if exhibiting brown cytoplasmic staining. Fisher’s exact test was used to compare the percentage of cases with cells expressing MMP-2 and MMP-9 in control and study groups (p < 0.05).

**Results:**

Light microscopy showed a higher concentration of cells with positive cytoplasmic staining for MMP-2 and MMP-9 expression in breast cancer than in fibroadenoma. The percentage of cases with cells expressing MMP-2 in the control and study groups was 41.67% and 86.11%, respectively (p < 0.0009), whereas the percentage of cases with cells expressing MMP-9 in groups A and B was 66.67% and 93.33%, respectively (p<0.0138). MMP-2 and MMP-9 positive expression was significantly higher in moderately differentiated tumors compared to well and poorly differentiated tumors, p <0.005 and p<0.001, respectively.

**Conclusions:**

The current study shows that MMP-2 and MMP-9 protein expression was significantly higher in the breast cancer than in the fibroadenoma and also in moderately differentiated breast cancer.

## INTRODUCTION

Breast cancer is the most common malignancy that affects women worldwide [1]. This disease is the second most frequently found cancer in the female population of Brazil, and 59,700 new cases and 14,206 deaths have been estimated in the year 2018 [2]. Although early detection and targeted therapy of breast cancer have improved disease prognosis [3], it has been suggested that more adequate therapeutic and prognostic strategies for breast cancer can be formulated with the use of protein biomarkers such as cell proliferation and apoptosis, since they have the advantage of suffering modifications before any clinical response of the tumor to treatment and may suggest changes in the therapy [4–6]. Biomarkers as matrix metalloproteinases, a family of zinc-dependent endopeptidases, whose major members of the family are MMP-2 and MMP-9 have been related to the pathogenesis of breast cancer [7, 8].

These proteinases have aroused the interest of researchers due to their association with cell proliferation, capacity to degrade collagen IV found primarily in the basal lamina, and thus favoring the migration of malignant cells. Furthermore, metalloproteinases are correlated with angiogenesis, which is essential for tumor growth and formation of metastases [9–11]. Studies have showed that concentrations of MMP-2 and MMP-9 are increased in women with breast cancer, and are associated with an unfavorable prognosis [12–14]. In addition, the literature has shown increased expression of MMP-2 and -9 in breast cancer in women with lymph node metastasis as compared to the expression of these gelatinases in breast cancer without metastases in axillary lymph nodes [15–18].

According to Ciurea et al. [19], fibroadenomas are the most common benign lesions in young women of reproductive age and some authors do not consider fibroadenoma as a tumor, preferring to label it as an aberration of normal development and involution (ANDI) [20]. Likewise, this consideration of the fibroadenoma as an alteration of normal breast tissue allows the use of samples from this tumor as control group for evaluating the expression of biomarkers in breast cancer such as metalloproteinases. Therefore, the prevalence of fibroadenoma of the breast and breast cancer in reproductive-aged women, the role of matrix metalloproteinase as a potential marker of tumor development and aggressiveness, in addition to the paucity of studies evaluating MMP expression in fibroadenoma and breast cancer, motivated us to create the design of current study.

## RESULTS

**Figure 1:**
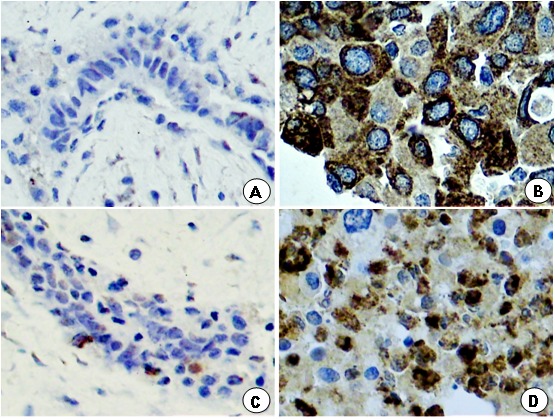
Photomicrography of histological section of a portion of fibroadenoma and breast cancer, showing: **(B, D)** innumerous cells with strong brown-stained cytoplasm for MMP-2 and MMP-9 in the breast cancer group compared to sparse brown stained cytoplasm in the breast fibroadenoma group **(A, C)**.

Light microscopy showed greater concentration of stained cytoplasm for MMP-2 and MMP-9 in breast cancer in comparison to fibroadenoma (Figure 1). The characteristics of both groups were similar, except for age and waist circumference (Table 1). Concerning quantification of immunohistochemical antigen expression, the percentage of positive cases for MMP-2 in group A (fibroadenoma) and B (breast cancer) was 15 (41.67%) and 25 (86.11%), respectively (p<0.0009), while the percentage of positive cases for MMP-9 in breast tumor tissues of women from groups A and B was 25 (66.67%) and 28 (93.33%), respectively (p < 0.0138) (Table 2). Regarding the histological grade, the breast cancer group showed positive expression for MMP-2 in 13 (52%) moderately differentiated (histological grade II) tumors, 10 (40%) histological grade III tumors and 2 (8%) histological grade I tumors. MMP-2 positive expression was significantly higher in moderately differentiated tumors compared to well and poorly differentiated tumors (p <0.005). MMP-9 expression was positive in 14 (50%) histological grade II tumors, 12 (43%) histological grade III tumors and 2 (7%) histological grade I tumors and was significantly higher in histological grade II than Grade I and III (p <0.001).

**Table 1:** Characteristics of the patients

	Group A (Fibroadenoma) (n=36) Mean±SD	Group B (Breast Cancer) (n=30) Mean±SD	p
Age (years)	32.92 ± 9.46	40.37 ± 6.77^*^	0.0011
Menarche age (yrs)	12.86 ± 1.16	13.67 ± 1.79	0.0820
BMI (kg/m^2^)	24.23 ± 4.79	25.83 ± 3.65	0.1342
WC (cm)	79.40 ± 12.34	83.92 ± 8.92^*^	0.0480

^*^ There was a statistically significant differences between control and study groups (p<0.05).

**Table 2:** Percentage of cases with cells expressing MMP-2 and MMP-9 in the breast fibroadenoma (Group A, control) and in the breast cancer (Group B, study)

Variable	Groups	Positive n (%)	Negative n (%)	Total n (%)
MMP-2	Fibroadenoma Breast cancer	15 (41.67%) 25(86.11%)^*^	21 (58,33%) 5 (13.89%)	36 (100.00) 30 (100.00)
MMP-9	Fibroadenoma Breast cancer	24 (66.67%) 28 (93.33%)^*^	12 (33,33%) 2 (6.67%)	36 (100.00) 30 (100.00)

^*^ The difference of MMP-2 and MMP-9 expression in the breast cancer group was statistically higher in comparison to fibroadenoma, p <0.0009 and p<0.0138, respectively.

## DISCUSSION

Matrix metalloproteinases have increased proteolytic activity against the basement membrane, leading to propagation of malignant cells [21]. The present study evaluated MMP-2 and MMP-9 expression in breast cancer cells in comparison to breast fibroadenoma, considered by some authors as only an aberration of normal development and involution (ANDI) [20] and showed heterogeneity between the age of breast cancer patients and age of women with fibroadenoma. However, fibroadenoma is more common in younger women, while breast carcinoma is more frequently found in older women [22]. Waist circumference was larger in breast cancer patients which agrees with findings in literature, where some authors believe that premenopausal women with excessive visceral fat are at increased risk of developing triple-negative breast cancer, a more aggressive tumor [23].

The current study showed a higher concentration of cells with strong brown cytoplasmic staining for MMP-2 and MMP-9 in breast cancer cells than in fibroadenoma. Matrix metalloproteinases expression has been shown in both breast normal tissue fibroadenoma and breast cancers [8], nevertheless, there is a scarcity of studies in the literature comparing the immunohistochemical expression of these gelatinases between breast cancer and fibroadenoma. To the best of our knowledge, only one study in the literature evaluated the immunohistochemical expression of MMP-2 in breast cancer and fibroadenoma and showed that a high expression of MMP-2 in breast ductal carcinoma *in situ* was an early incident in the genetic course of the breast cancer [24]. On the other hand, the majority of studies in literature have investigated serum concentrations of MMP-2 and MMP-9 and showed a higher concentration of these serum proteins in breast cancer patients than in women with fibroadenoma, corroborating data of immunohistochemical expression [15, 16, 25].

MMP-2 and MMP-9 participate in both early and late processes of tumor progression [26]. It is noteworthy that MMP activity may be direct through tumor proliferation and metastatic dissemination by degradation of the extracellular matrix and basement membrane. MMP activity may also be indirect by promoting angiogenesis [27]. MMP-9 in particular, has a significant role in this process by activating vascular endothelial growth factor (VEGF), which is one of the major contributors to the formation of new vessels and tumor growth [28].

Huang et al. [15] evaluated the expression of MMP-2 and MMP-9 in breast cancer tissue and serum of women with cancer and those with benign breast tumors, showing that 72% and 76% of women with breast cancer had a positive expression for MMP-2 and MMP-9, respectively. The authors also demonstrated higher serum levels of these metalloproteinases in breast cancer patients when compared to women with benign tumors. Sullu et al.[12] and Min et al. [17], also analyzed the immunohistochemical expression of MMP-2 and MMP-9 in invasive ductal carcinoma and identified a strong cytoplasmic staining for MMP-9 in 66% and 93.8% of cases, respectively.

Therefore, our results are in accordance with findings by Li et al. [7] and Min et al. [17] who showed a significantly higher expression of MMP-2 and MMP-9 in breast cancer than in normal adjacent tissue and grade II cancers, respectively. This suggests that these metalloproteinases are potentially associated with tumor aggressiveness and may be a prognostic biomarker of breast cancer. To the best of our knowledge, there is scarcity of studies evaluating the immunohistochemical expression of MMP-2 and MMP-9 in breast cancer and breast fibroadenoma. Nevertheless, Sampaio et al. [29] recently showed significantly higher expression of metallotionein-1, a zinc-linked protein, in breast cancer than in fibroadenoma. Thus, due to the paucity of studies in the literature, further studies with a larger sample size are necessary to improve knowledge of the role of MMP-2 and MMP-9 in the progression and prognosis of breast cancer.

## PATIENTS AND METHODS

### Patients

The study was approved by Review Board of the Federal University of Piaui (CAAE: 43447015.8.0000.5214) and all the patients signed an informed consent term prior to the beginning of the study. In addition, we confirm that all methods were performed in compliance with current Brazilian laws, in conformity with ethical standards of institutional and national research committees, following the 1964 Helsinki Declaration and its later amendments. Patients were recruited at the Mastology Clinic of the Getúlio Vargas Hospital, Federal University of Piauí, Brazil, from October 2014 to October 2016. The study included premenopausal patients with levels of follicle-stimulating hormone (FSH) < 30 mUI/ml, fibroadenoma or carcinoma of the breast and no previous oncologic treatment.

### Study design

A randomized, double-blind study was carried out, involving 75 premenopausal women with breast tumors. Nine patients were excluded due to technical problems that precluded analysis. Patients were divided into two groups, control group (fibroadenoma, n = 36) and study group (breast cancer, n = 30). All study participants underwent a specialized surgical procedure for histologic and immunohistochemical confirmation of the tumor. Malignant tumors were classified as poorly differentiated (Grade III), moderately differentiated (Grade II) and well-differentiated (Grade I) with a low degree of malignancy.

### Immunohistochemistry of MMP-2 and MMP-9

For immunohistochemical analysis, breast tissue fragments were fixed in buffered formalin, cut into 3-μm thick sections, deparaffinized in xylol for 5 minutes, dehydrated in absolute ethanol, washed in buffered saline solution at pH 7.4 for 5 minutes and then treated for 5 minutes with 3% hydrogen peroxide (H_2_O_2_) in buffer to block endogenous peroxide.

For antigen retrieval, the slides were placed in racks containing 0.21% of citric acid (pH 6.0) and heated in a microwave oven at maximum power for 15 minutes. A buffered saline solution with phosphate containing Tween (PBS-Tween) was added to the slides after they were allowed to cool for 20 minutes. Tissue samples were incubated overnight at 4-8 °C with primary monoclonal antibody of the rat NCL-MMP2-507 and NCL-MMP9-439 (dilution of 1:50). The slides were washed with PBS-Tween, instilled with secondary reagent (anti-mouse BA 2000, Vector Laboratories, Burlingame, CA) and incubated for 30 minutes at room temperature. After washing once again with PBS-Tween, the slides were instilled with ABC Elite detection system (PK 6100, Vector Laboratories) and incubated for 45 minutes at room temperature.

Samples were once again washed with PBS-Tween, instilled with DAB (1.0 ml of EnVision FLEX DAB to one drop of chromogen) and incubated for 5 minutes. Finally, the slides were rinsed in distilled water, counterstained with hematoxylin, stained with ammoniacal silver solution, dehydrated with absolute ethanol, passed through Coplin jars containing xylol and mounted on Permount resin. Cells that expressed proteins MMP-2 and MMP-9 were identified by brown cytoplasmic staining.

### Quantitative method

Quantification was performed by two observers blinded to patient identity who were also previously unaware of the cases. The procedure was performed using a light microscope (Eclipse E-400 optical microscope, Nikon, Tokyo, Japan) connected to a video camera (CHC-370 N digital camera, Samsung, Seoul, Korea), that captured and transmitted the image to a computer equipped with Image Lab software, version 2.3, developed by Softium Informatica (São Paulo, Brazil) for image analysis.

A semi-quantitative analysis of MMP-2 and MMP-9 immunoreactivity was performed, according to criteria established by Van Slooten et al. [30]. The following parameters were taken into consideration: intensity of cell staining (I) and fraction of stained neoplastic cells (F). Staining intensity was graded as: 0 (negative), 1 (weakly stained), 2 (moderately stained) or 3 (strongly stained). The fraction of stained cells was classified as follows: I (0 - 25%), II (25 - 75%) or III (75 - 100%). The final result was achieved by a combination of two parameters (I and F) ranging from 0 to 6. Cases with a final score ≥ 3 were classified as positive for MMP-2 and MMP-9. In all cases, brown cytoplasmic staining was adopted as a pattern of positivity.

### Statistical analysis

Data were analyzed using SPSS statistical program for Windows 8.0. A comparison of age between both groups was analyzed by the Mann-Whitney nonparametric test [31], while Fisher’s exact test was used to assess Body Mass Index (BMI) and Waist Circumference (WC). The percentage of cases expressing MMP-2 and MMP-9 between both groups was analyzed by Fisher’s exact test. The expression of MMP 2 and MMP 9 in histological grades of breast cancer was analyzed by Friedman test. The significance level was set at p < 0.05 [32].
